# Whole-genome sequencing of the Streptomyces coelicolor bldA39 mutant (J1700) reveals hundreds of previously unknown mutations

**DOI:** 10.1099/acmi.0.000958.v3

**Published:** 2025-02-14

**Authors:** Jack W. Stone, John T. Munnoch, Paul A. Hoskisson

**Affiliations:** 1 Strathclyde Institute of Pharmacy and Biomedical Sciences, University of Strathclyde, Glasgow, 161 Cathedral Street, G4 0RE, UK

**Keywords:** antibiotic, *bldA*, development, morphological mutant, sporulation, *Streptomyces*

## Abstract

We report the genome sequence of the *bldA39* (J1700) mutant of *Streptomyces coelicolor*, a historically important strain that is deficient in sporulation and antimicrobial production. The *S. coelicolor* J1700 strain was used extensively from the 1980s onwards to underpin important discoveries in development and antibiotic production in *Streptomyces*. The *bldA* gene encodes a leucyl tRNA, required for the translation of the rare TTA codon found in ~2% of genes in *Streptomyces*. The whole genome of *S. coelicolor* J1700 was obtained via Illumina sequencing and mapped to the *S. coelicolor* M145 reference genome. Analysis of the genome sequence compared to *S. coelicolor* M145 identified the known *bldA39* mutation (T>C) and revealed more than 300 further mutations, likely associated with the *S. coelicolor* J1501 genetic background the strain was created in, including the nature of the *hisA1* and *uraA1* alleles used extensively in genetic mapping experiments and several mutations in natural product biosynthetic gene clusters. This work highlights the importance of whole-genome sequencing of historically important strains.

## Data Summary

This whole-genome sequencing project has been deposited in NCBI under the Bioproject PRJNA1186139. The WGS reads used can be accessed in the NCBI’s SRA under the accession number SAMN44744323. Table S1 is available on Figshare 10.6084/m9.figshare.27798405[1].

## Introduction

The bacterial genus *Streptomyces* has long been studied as a model for morphological differentiation and the production of natural products such as antibiotics [2]. Decades of genetic analysis of *Streptomyces* bacteria have enabled the identification of regulatory mechanisms that are essential for morphological development (formation of unigenomic spores on reproductive structures called aerial hyphae) and antibiotic production [3,5]. During these studies, numerous mutants have been isolated that are blocked at distinct stages of development, and these fall into two main classes: the so-called white (*whi*) mutants, which are able to form aerial mycelium but are unable to complete development into mature spores. The second class are the so-called bald (*bld*) mutants, which are blocked at an earlier stage of development, which prevents the erection of the aerial hyphae and subsequent development of spores. In addition to causing the loss of aerial mycelium, several mutations in *bld* loci have been found to pleiotropically block antibiotic production [6,7].

Amongst the most severe *bld* phenotypes that have been identified to date were associated with the *bldA* locus, where mutations result in complete loss of morphological development and natural product production [8,9]. The *bldA* locus was the first morphological mutant mapped by Hopwood [10] as *bldA1* (S48), with further mapping efforts of Merrick [11] characterizing 12 *bld* mutants into 4 mapping groups, 5 of which were *bldA* alleles. The *bldA* locus was cloned by Piret and Chater [12] and subsequently shown to encode a leucyl tRNA, required for the translation of the rare TTA codon found in ~2% of genes in *Streptomyces* [13,14]. The Leu-tRNA^UUA^ accumulates late in growth [13,15, 16], with much of the *bldA*-associated phenotype believed to be mediated via the highly conserved, TTA-codon containing global transcriptional regulator, AdpA [7,17]. The effect that *bldA* disruption has on the control of antibiotic production has also been attributed to TTA codons present in biosynthetic gene cluster (BGC) situated regulators such as *actII-ORF4*, *redD* (in *Streptomyces coelicolor* [18,19]) and *ccaR* (in *Streptomyces clavuligerus* [20]).

Amongst the original *bldA* mutants characterized by Merrick [11] was the *bldA39*, a mutation that was subsequently used in phage cloning experiments to transfer the mutation [12] to the *S. coelicolor* J1501 strain background (*his1A*, *ura1A*, *strA1*, *pgl-1*, SCP1^−^, SCP2^−^ [21]) that was historically used for genetic mapping experiments. This created the *S. coelicolor* J1700 (*bldA39*) strain that was subsequently used in studies by Leskiw *et al*. [14,16] to characterize the *bldA* gene. The genetic lesion leading to the *bldA* morphological phenotype can be complemented through the addition of a copy of the *bldA* gene on a phage [12] and by integrating plasmids (Stone, Munnoch and Hoskisson, unpublished). Studies of antibiotic production in the *S. coelicolor* J1700 (*bldA39*) strain found that there is reduced expression of genes in the undecylprodigiosin (*red*) BGC [21]. Actinorhodin (*act*) production appears to be predominantly regulated at the level of transcription, although translation fusions of the 5′ end of *actII-ORF4* containing a single UUA codon to an *ermE* gene demonstrated that the *bldA* tRNA is present and functional early in growth [22].

Many of the studies to date on *bldA* have been conducted in the *S. coelicolor* J1700 (*bldA39*) strain; however, the wider genetic background of this strain is currently unknown. Here, we describe the genome sequencing of the *S. coelicolor* J1700 mutant and provide further information on additional mutations in that strain background. These data are deposited in NCBI under the Bioproject PRJNA1186139. The WGS reads (paired-end Illumina data) used can be accessed in the NCBI’s SRA under the accession number SAMN44744323.

## Methods

*S. coelicolor* J1700 was grown for 24 h in Tryptone Soy Broth (TSB) media at 30 °C, shaking at 200 r.p.m. The genomic DNA of the strain was extracted according to Kieser *et al*. [23], and modifications were provided in Actinobase [24]. Sequencing was performed by Novogene using the Illumina NovaSeq 6000 platform. DNA sequence analysis enabled the mapping of the reads to the *S. coelicolor* M145 chromosome [25]. Breseq [26] mapping analysis of each strain was performed (using default settings, without predict-polymorphisms) and the output GenomeDiff files were compared (gdtools COMPARE). The analysis reports ‘predicted mutations’, including small variants (indels and single nucleotide changes), regions of ‘unassigned missing coverage evidence’ (typically large deletions) and ‘unassigned new junction evidence’ where multiple forms of the same sequence are suggested by the data (typically deletions with read coverage of the *S. coelicolor* M145 reference sequence also present). Mutations were then transferred to the reference genome (using gdtools APPLY) generating a FASTA, GENBANK and GFF3 version of the genome. This was carried out for all mutations in ‘predicted mutations’ while necessary manual edits were made as required. Auxotroph analysis was carried out according to Kieser *et al*. [23].

## Results & discussion

### *S. coelicolor* J1700 has extensive mutations across the genome that likely reflect the genotype of the parental strain J1501

**Fig. 1.**
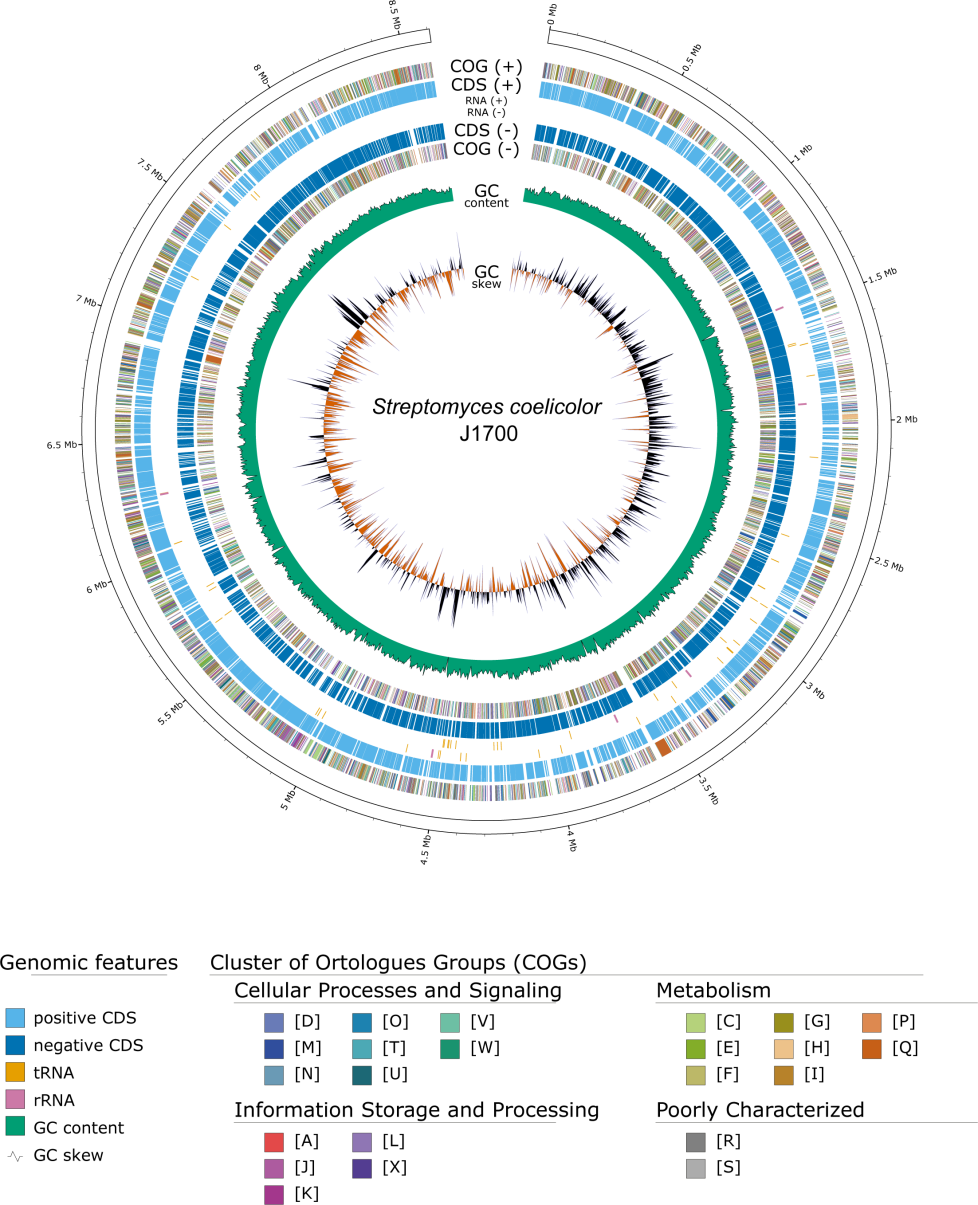
GenoVi visualization of the *S. coelicolor* J1700 genome [35]. Labelling from outside to the inside: COGs [36] (forward strand); CDS, tRNAs and rRNAs (forward strand); CDS, tRNAs and rRNAs (reverse strand); COGs (reverse strand); Genome G+C content; Genome GC skew.

The whole-genome sequence of *S. coelicolor* J1700 was determined at 137.6× coverage and was mapped to the wild-type *S. coelicolor* M145 strain (NC_003888.3) [22] (Fig. 1). The *S. coelicolor* J1700 strain was originally constructed in the *S. coelicolor* J1501 genetic background (*his1A, ura1A, strA1, pgl-1,* SCP1^−^, SCP2^−^) that was historically used for genetic mapping experiments [23]. The *S. coelicolor* J1700 strain was created through phage-mediated transfer of the *bldA39* mutation [12] to *S. coelicolor* J1501, although the overall genetic background of the strain remains unknown.

The genome of *S. coelicolor* J1700 was found to be 8 608 660 bp (Fig. 1), consisting of 7823 CDSs (compared to the 7846 CDSs in *S. coelicolor* M145 [25]). Following the Breseq analysis, an ‘unassigned missing coverage evidence’ region of the *S. coelicolor* J1700 genome was identified, which indicates a 53 414 bp deletion between 7 014 046 bp and 7 071 460 bp of the genome. This deletion results in the loss of the *SCO6353-SCO6406* genes. Comparison with *S. coelicolor* M145 reveals that there are 324 mutations in J1700 (Fig. 1 and Table S1: 10.6084/m9.figshare.27798405, available in the online Supplementary Material). The mutations in *S. coelicolor* J1700 are characterized as 121 non-synonymous mutations, 78 synonymous mutations, 74 intergenic mutations, 39 coding frameshifts, 3 pseudogenes (SCO0634, SCO2890 and SCO4318), 3 deletions of ~1 kb (affecting SCO3991–SCO3991, [SCO4697]–[SCO4699] and SCO5630–[SCO5632], where the square brackets indicate a potential polar affect on that gene), 3 non-sense, 2 non-coding (including *bldA* and methionine tRNA anticodon CAT) and 2 non-stop mutations. It is likely that many of these mutations reflect those in the *S. coelicolor* J1501 genetic background in which J1700 was constructed [12].

### The *bldA39* mutation in *S. coelicolor* J1700 results in an anticodon change from Leu-UAA to Ser-UGA

Lawlor *et al*. [13] first showed that the *bldA39* mutation results in a mutation in the anticodon loop of the Leucyl-tRNA^UUA^, which generates a putative seryl-anticodon. It is currently unclear if this tRNA species can be charged with serine by the cognate aminoacyl-tRNA synthetase (aaRS). Given the selectivity of aaRSs enzymes, this is unlikely as there are limited editing mechanisms in the aaRSs between the cognate tRNAs for serine and leucine [27]. The *bldA39* mutant represents the only ‘classical’ *bldA* mutant strain that disrupted the tRNA anticodon, with other mutations affecting the anticodon stem of the tRNA*^bldA^* (*bldA1* [nt 28 G-A]) and the tRNA*^bldA^* d-arm (*bldA16* [nt 22 C-T], *bldA62* [nt 23 A-C]) [21]. The single nucleotide T-C mutation attributed to the *bldA39* phenotype is found at position 3 380 959 in *S. coelicolor* J1700 chromosome (position 3 380 943 in *S. coelicolor* M145).

### Discrepancies in undecylprodigiosin expression on *S. coelicolor* J1700 may be the result of *IS110* located in the BGC (*red*)

AntiSMASH [28] of the *S. coelicolor* J1700 genome revealed the presence of all 24 BGCs known from *S. coelicolor* M145. A detailed investigation of the BGCs indicated that there are several mutations within these gene clusters.

Guthrie and Chater [21] reported reduced *red* gene expression in the *S. coelicolor* J1700 strain using *xylE* transcriptional reporter strains. Examination of the *S. coelicolor* J1700 reveals the presence of a synonymous mutation in the undecylprodigiosin BGC pathway-specific regulator *redD* [19] (SCO5877: CTC-CTT; L150L). This reflects a change to a much less frequently used codon, but which is unlikely to impact significantly on *red* gene expression. More likely to affect transcription of the *red* cluster in *S. coelicolor* J1700 is the presence of an *IS110* element [29] in the intergenic region between SCO5885 (putative membrane protein) and SCO5886 (*redR*, which encodes a 3-oxoacyl-[acyl-carrier protein] synthase II) at position 6 442 702 bp in the genome.

Further mutations in BGCs were noted, such as in the coelichelin BGC, with a synonymous mutation in a putative peptide synthetase (SCO0492; TTC-TTT; F2247F). Two non-synonymous mutations were noted in the calcium-dependent antibiotic (CDA) BGC in the CDA peptide synthetase I (SCO3230; CTC-GTC; L3479A; and GCC-GTC; A5927V). A non-synonymous mutation was also identified in the actinorhodin (*act*) BGC, in the ActIV bifunctional cyclase [second ring] thioesterase [30] (SCO5091; GCG-GAC; A689D). Additional mutations are also present in the coelimycin BGC [31], where two synonymous mutations are present in *cpkPβ* (SCO6269: GCG-GCC; A179A; and GCG-GCA; A166A) and two further synonymous mutations in *cpkC* (SCO6273: AAG-AAA; K562K; and GGG-GGC; G561G).

The consequences of these mutations are unknown; however, with mostly synonymous mutations present in the BGC genes, there are unlikely to be significant effects on the phenotype of *S. coelicolor* J1700, with extensive complementation studies required to assess potential effects on phenotype and through mRNA stability where synonymous changes are present.

### The *hisa1* genotype is a result of mutation in the histidinol dehydrogenase gene, *hisD*

**Fig. 2.**
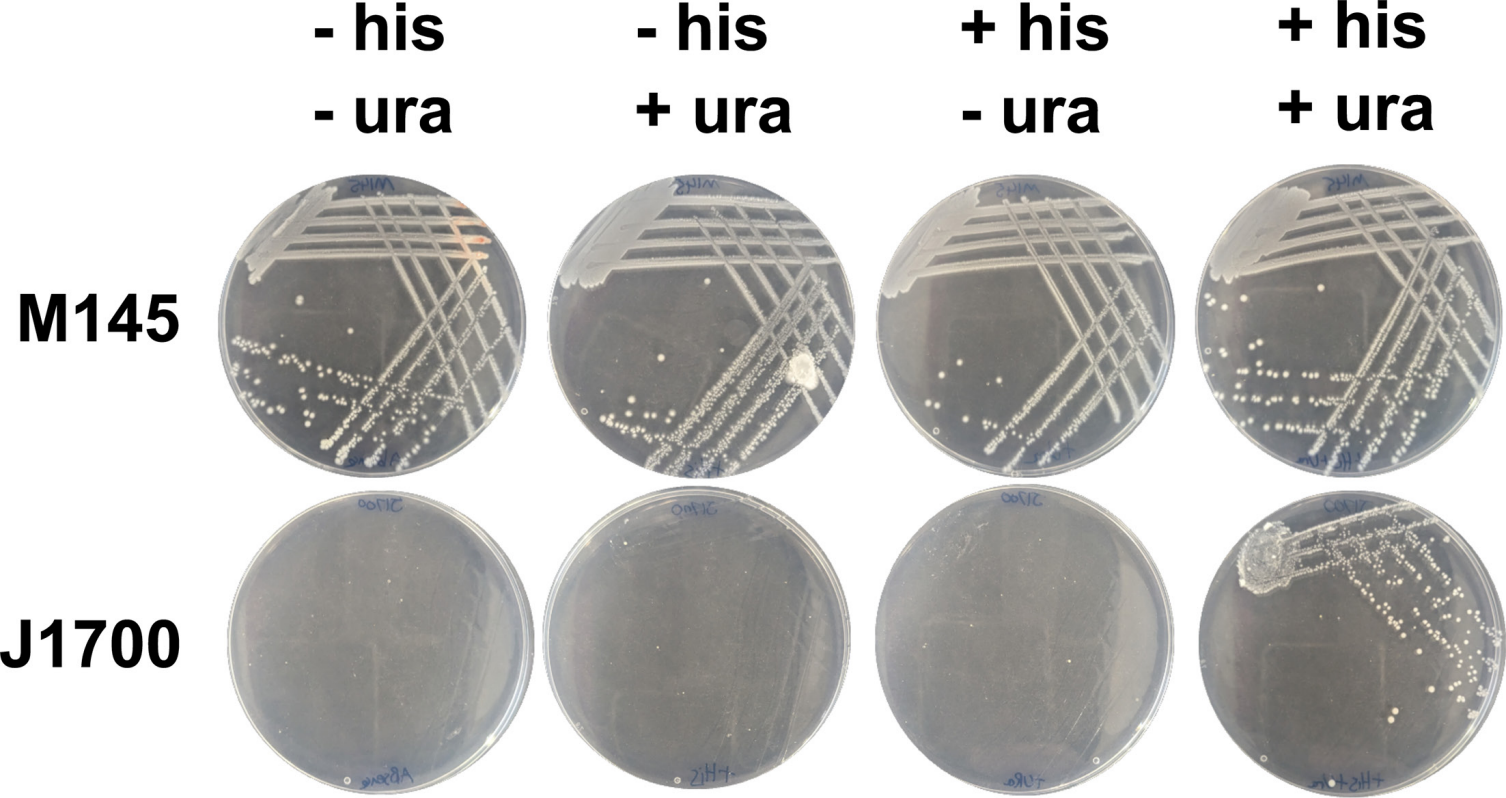
Auxotrophic analysis of the *S. coelicolor* J1700 strain. The *S. coelicolor* wild-type (M145) and *bldA39* (J1700) strain were grown in the presence of histidine (his) or uracil (ura) according to Kieser *et al*. [23] to test for auxotrophy based on the genotype of the parental strain *S. coelicolor* J1501.

One of the genetic markers present in *S. coelicolor* J1501 strain, the progenitor of the *bldA*39 strain J1700, is *hisA1*. Strains carrying this mutation are histidine auxotrophs [23]. The designation of *hisA1* as a mapping group is well established, but the literature is not clear about where the mutation that results in histidine auxotrophy is situated. This may reflect the use of ‘*hisA’* a complementation group in older work on *S. coelicolor* genetics. Work from Limauro *et al*. [32] suggests that the so-called *hisA* gene in *S. coelicolor* was in fact an ortholog of *hisD*, the histidinol dehydrogenase in *Escherichia coli*. Histidinol dehydrogenase catalyses the terminal reaction in histidine biosynthesis that oxidizes l-histindol to l-histidine and in *S. coelicolor* is the first gene in a three-gene operon (*hisDCB*). Sequencing of *S. coelicolor* J1700 identified a missense mutation in the gene *hisD* (T-C) resulting in an E264G change in histidinol dehydrogenase. This mutation maps to the region of the protein that coordinates a catalytic zinc ion that is required for substrate binding [33]. To confirm the requirement of *S. coelicolor* J1700 for histidine, growth on minimal media was tested for its ability to support *S. coelicolor* J1700 in the presence and absence of histidine, confirming auxotrophy (Fig. 2).

### The *uraA1* mutation maps to the putative uridine 5′-monophosphate synthase in *S. coelicolor* J1700

A further historic and widely used genetic marker in *S. coelicolor* J1501 is *uraA1*, where strains exhibit uracil auxotrophy. Analysis of the mutations detected in *S. coelicolor* J1700 revealed that there was no mutation present in the *uraA* gene, suggesting that this may also reflect the use of *uraA* as a complementation group designation rather than a gene designation. Auxotrophy analysis of the strain revealed *S. coelicolor* J1700 is auxotrophic for uracil (Fig. 2). Analysis of the genome mutations in *S. coelicolor* J1700 identified a putative uridine 5′-monophosphate synthase (SCO3650: *pyrE*) that possesses a frameshift mutation resulting in a 10 bp deletion (99–108/549 nt) towards the 5′ end of the CDS. The *pyrE* gene also maps to the *uraA1* location of the physical map of the *S. coelicolor* chromosome [34], suggesting that it is this mutation that results in uracil auxotrophy in *S. coelicolor* J1700. Uridine 5′-monophosphate synthases catalyse the formation of uridine monophosphate as an initial step in uridine triphosphate biosynthesis and RNA metabolism. This led us to conclude that uracil auxotrophy is likely the result of a frameshift in *pyrE* of *S. coelicolor*.

## Summary

The whole-genome sequencing of bacterial strains has revolutionized the way microbiology is conducted. The sequencing of historical strains that have underpinned significant discoveries in particular fields can help to shed light on discrepancies in older literature, such as here around discrepancies in *red* gene expression in *S. coelicolor* J1700, that could be attributed to the presence of a previously discovered insertion element. Furthermore, the clarification of genetic markers that were historically used to map mutations can help clarify the literature for researchers who may never have undertaken genetic mapping experiments. Overall, the sequencing of the historically important *S. coelicolor* J1700 *bldA39* strain will provide a resource for researchers to use in studying development and antibiotic production in *Streptomyces*.

## Abbreviations

aaRS: aminoacyl-tRNA synthetase
BGC: biosynthetic gene cluster
CDA: calcium-dependent antibiotic
TSB: tryptone soy broth

## References

[1] Stone J, Munnoch J, Hoskisson PA (2024). *Figshare*.

[2] Schlimpert S, Elliot MA (2023). The best of both worlds—*Streptomyces coelicolor* and *Streptomyces venezuelae* as model species for studying antibiotic production and bacterial multicellular development. J Bacteriol.

[3] Chater KF (1998). Taking a genetic scalpel to the *Streptomyces* colony. Microbiology.

[4] Bush MJ, Tschowri N, Schlimpert S, Flärdh K, Buttner MJ (2015). c-di-GMP signalling and the regulation of developmental transitions in streptomycetes. Nat Rev Microbiol.

[5] Flärdh K, Buttner MJ (2009). *Streptomyces morphogenetics*: dissecting differentiation in a filamentous bacterium. *Nat Rev Microbiol*.

[6] Chandra G, Chater KF (2014). Developmental biology of *Streptomyces* from the perspective of 100 actinobacterial genome sequences. FEMS Microbiol Rev.

[7] Chandra G, Chater KF (2008). Evolutionary flux of potentially bldA-dependent *Streptomyces* genes containing the rare leucine codon TTA. Antonie van Leeuwenhoek.

[8] Chater KF, Chandra G (2006). The evolution of development in Streptomyces analysed by genome comparisons. FEMS Microbiol Rev.

[9] Hackl S, Bechthold A (2015). The gene blda, a regulator of morphological differentiation and antibiotic production in *Streptomyces*. Arch Pharm.

[10] Hopwood DA (1967). Genetic analysis and genome structure in *Streptomyces coelicolor*. Bacteriol Rev.

[11] Merrick MJ (1976). A morphological and genetic mapping study of bald colony mutants of *Streptomyces coelicolor*. J Gen Micro.

[12] Piret JM, Chater KF (1985). Phage-mediated cloning of bldA, a region involved in *Streptomyces coelicolor* morphological development, and its analysis by genetic complementation. J Bacteriol.

[13] Lawlor EJ, Baylis HA, Chater KF (1987). Pleiotropic morphological and antibiotic deficiencies result from mutations in a gene encoding a tRNA-like product in *Streptomyces coelicolor* A3(2). Genes Dev.

[14] Leskiw BK, Bibb MJ, Chater KF (1991). The use of a rare codon specifically during development?. Mol Microbiol.

[15] Leskiw BK, Mah R, Lawlor EJ, Chater KF (1993). Accumulation of bldA-specified tRNA is temporally regulated in *Streptomyces coelicolor* A3(2). J Bacteriol.

[16] Leskiw BK, Lawlor EJ, Fernandez-Abalos JM, Chater KF (1991). TTA codons in some genes prevent their expression in a class of developmental, antibiotic-negative, *Streptomyces* mutants. Proc Natl Acad Sci USA.

[17] Takano E, Tao M, Long F, Bibb MJ, Wang L (2003). A rare leucine codon in adpA is implicated in the morphological defect of bldA mutants of *Streptomyces coelicolor*. Mol Microbiol.

[18] Liu G, Chater KF, Chandra G, Niu G, Tan H (2013). Molecular regulation of antibiotic biosynthesis in *Streptomyces*. Microbiol Mol Biol Rev.

[19] Malpartida F, Niemi J, Navarrete R, Hopwood DA (1990). Cloning and expression in a heterologous host of the complete set of genes for biosynthesis of the *Streptomyces coelicolor* antibiotic undecylprodigiosin. Gene.

[20] Trepanier NK, Jensen SE, Alexander DC, Leskiw BK (2002). The positive activator of cephamycin C and clavulanic acid production in *Streptomyces clavuligerus* is mistranslated in a bldA mutant. Microbiology.

[21] Guthrie EP, Chater KF (1990). The level of a transcript required for production of a *Streptomyces coelicolor* antibiotic is conditionally dependent on a tRNA gene. J Bacteriol.

[22] Gramajo HC, Takano E, Bibb MJ (1993). Stationary-phase production of the antibiotic actinorhodin in *Streptomyces coelicolor* A3(2) is transcriptionally regulated. Mol Microbiol.

[23] Kieser BM, Bibb M, Buttner M, Chater K, Hopwood D (2000). Practical Streptomyces Genetics.

[24] Feeney MA, Newitt JT, Addington E, Algora-Gallardo L, Allan C (2022). ActinoBase: tools and protocols for researchers working on *Streptomyces* and other filamentous actinobacteria. *Microb Genom*.

[25] Bentley SD, Chater KF, Cerdeño-Tárraga A-M, Challis GL, Thomson NR (2002). Complete genome sequence of the model actinomycete *Streptomyces coelicolor* A3(2). Nature.

[26] Deatherage DE, Barrick JE (2014). Identification of mutations in laboratory-evolved microbes from next-generation sequencing data using breseq. Methods Mol Biol.

[27] Tawfik DS, Gruic-Sovulj I (2020). How evolution shapes enzyme selectivity - lessons from aminoacyl-tRNA synthetases and other amino acid utilizing enzymes. FEBS J.

[28] Blin K, Shaw S, Kloosterman AM, Charlop-Powers Z, Wezel GP van (2021). antiSMASH 6.0: improving cluster detection and comparison capabilities. Nucleic Acids Res.

[29] Chater KF, Bruton CJ, Foster SG, Tobek I (1985). Physical and genetic analysis of IS110, a transposable element of *Streptomyces coelicolor* A3(2). *Mol Gen Genet*.

[30] Taguchi T, Awakawa T, Nishihara Y, Kawamura M, Ohnishi Y (2017). Bifunctionality of ActIV as a cyclase‐thioesterase revealed by *in vitro* reconstitution of actinorhodin biosynthesis in *Streptomyces coelicolor* A3(2). Chembiochem.

[31] Gomez-Escribano JP, Song L, Fox DJ, Yeo V, Bibb MJ (2012). Structure and biosynthesis of the unusual polyketide alkaloid coelimycin P1, a metabolic product of the cpk gene cluster of *Streptomyces coelicolor* M145. Chem Sci.

[32] Limauro D, Avitabile A, Cappellano C, Puglia AM, Bruni CB (1990). Cloning and characterization of the histidine biosynthetic gene cluster of *Streptomyces coelicolor* A3(2). Gene.

[33] Barbosa JARG, Sivaraman J, Li Y, Larocque R, Matte A (2002). Mechanism of action and NAD+-binding mode revealed by the crystal structure of L-histidinol dehydrogenase. Proc Natl Acad Sci USA.

[34] Redenbach M, Kieser HM, Denapaite D, Eichner A, Cullum J (1996). A set of ordered cosmids and a detailed genetic and physical map for the 8 Mb *Streptomyces coelicolor* A3(2) chromosome. Mol Microbiol.

[35] Cumsille A, Durán RE, Rodríguez-Delherbe A, Saona-Urmeneta V, Cámara B (2023). GenoVi, an open-source automated circular genome visualizer for bacteria and archaea. PLoS Comput Biol.

[36] Galperin MY, Wolf YI, Makarova KS, Alvarez RV, Landsman D (2021). COG database update: focus on microbial diversity, model organisms, and widespread pathogens. Nucleic Acids Res.

